# From Point Clouds to Predictive Maintenance: A Review of Intelligent Railway Infrastructure Monitoring

**DOI:** 10.3390/s26041131

**Published:** 2026-02-10

**Authors:** Yalin Zhang, Peng Dai, Mykola Sysyn, Yuchuan Hu, Lei Kou, Haoran Song, Jing Shi

**Affiliations:** 1 Infrastructure Inspection Research Institute, China Academy of Railway Sciences, Beijing 100081, China; 202435474@mail.sdu.edu.cn (Y.Z.); 13659354500@163.com (H.S.); 15536364887@163.com (J.S.); 2 School of Qilu Transportations, Shandong University, Jinan 250100, China; 202435516@mail.sdu.edu.cn; 3 Institute of Railway Systems and Public Transport, Technical University Dresden, 01069 Dresden, Germany; mykola.sysyn@tu-dresden.de

**Keywords:** point cloud technology, railway, monitoring and detection, data acquisition and processing, multi-domain fusion

## Abstract

Point cloud technology, characterized by its high-precision 3D geometric acquisition in complex railway environments, has become a cornerstone for the intelligent detection, monitoring, and maintenance of railway infrastructure. This paper provides a systematic review of point cloud applications across critical railway scenarios, encompassing track geometry extraction, infrastructure component identification, tunnel and bridge modeling, clearance and encroachment analysis, and structural condition monitoring. We evaluate various mobile and stationary acquisition platforms alongside their typical data processing workflows. Furthermore, this review synthesizes cutting-edge advancements in processing algorithms, with a focus on feature extraction, semantic segmentation, and the transformative impact of deep learning and artificial intelligence on data fusion. Notably, the paper explores the synergy between point clouds and computational mechanics, specifically the construction of high-fidelity digital twins through multi-physics coupling to enable real-time simulation of structural stress distribution and damage evolution. We critically analyze persistent technical bottlenecks, such as acquisition efficiency, monitoring precision, data fragmentation, environmental interference, and the complexities of multi-modal data fusion. Finally, the paper outlines future research trajectories, focusing on autonomous intelligent sensing, multi-sensor integration, and the comprehensive digital transformation of railway infrastructure management, aiming to provide a robust theoretical framework and technical roadmap for the sustainable intelligentization of global railway systems.

## 1. Introduction

In modern society, railways serve as a critical component of the national economic framework and significantly contribute to economic development, social stability, and the enhancement of living standards [1]. Ensuring their reliable operation is fundamental for efficient passenger and freight transport. However, traditional railway inspection and maintenance still largely depend on manual methods, which suffer from notable drawbacks [2]: low efficiency due to the vast network and long inspection cycles, high labor and operational costs, and safety risks for personnel working in hazardous environments. With increasing rail traffic and higher operational speeds, these conventional approaches are becoming inadequate for modern intelligent monitoring and maintenance needs. This highlights an urgent demand for lifecycle management and preventive maintenance strategies in railway infrastructure [3].

The emergence of point cloud technology has introduced new opportunities for the railway sector. The point cloud is composed of data points, each defined by x, y, and z coordinates that determine its spatial position. In certain cases, supplementary information may accompany these points, such as intensity, RGB color, or timestamp, which collectively characterize the sampled three-dimensional (3D) environment. Comprehensive geometric information of the sampled environment can be derived by integrating point clouds and establishing a unified coordinate system [4]. With core advantages of high precision, non-contact data acquisition and full element coverage, point cloud technology facilitates rapid and accurate collection of 3D data of railway infrastructure, thereby providing a solid data foundation for subsequent analysis and decision-making [5]. Moreover, the trend towards multidisciplinary integration has significantly enhanced the application of point cloud technology in the railway sector. The synergistic combination of photogrammetry, LiDAR, artificial intelligence (AI), and DT enhances the intelligence and performance in point cloud data collection, processing, and analysis. It also paves the way for innovative monitoring and maintenance solutions in railway infrastructure [6,7]. Currently, scholars worldwide have made notable achievements in the application of point cloud technology in the railway sector. Research efforts have focused on areas such as track status detection, catenary monitoring, topography and geology monitoring, foreign body intrusion monitoring, and bridge and tunnel health monitoring, yielding positive results including accurate measurements of track geometric parameters [8,9,10], automatic identification of catenary components [11,12], effective monitoring of geological disasters [13,14], and early warning of structural deformation in bridges and tunnels [15,16,17]. Nevertheless, existing technologies still have numerous limitations. In terms of dynamic monitoring accuracy, current methods struggle to meet high accuracy demands in the context of complex railway operation environments and frequently changing facility conditions. Moreover, the complex structure of railway infrastructure often hampers the identification of minor defects, resulting in low identification rates. Additional technical gaps exist in data acquisition and processing efficiency, real-time and generalization capabilities of algorithm, and cross-platform data interoperability, all of which require further exploration and resolution. Point cloud technology is crucial for railway digital transformation. High-precision 3D modeling and real-time monitoring of railway infrastructure foster the digital management of railway systems, thereby enhancing management efficiency and promoting scientific decision-making [18]. Furthermore, point cloud technology offers effective technical solutions to critical challenges within the railway industry, including comprehensive structure health monitoring for timely identification of safety hazards and support for preventive maintenance strategies. Additionally, it optimizes railway operation and maintenance strategies through an intelligent decision support system, thereby reducing operational costs. The research on point cloud technology in the railway sector has promoted interdisciplinary integration and opened new avenues for scholarly inquiry. In terms of engineering applications, its achievements directly enhance railway construction, operation, and maintenance, yielding significant economic and social benefits.

Therefore, a thorough investigation of point cloud technology applications in the railway sector is of practical significance. This study systematically reviews the current applications of point cloud technology in key railway scenarios, analyzes the technical challenges, and anticipates future development directions. The aim is to provide theoretical support and practical reference for the intelligent upgrading of railway infrastructure while promoting the sustainable development of railway industry.

**Figure 1:**
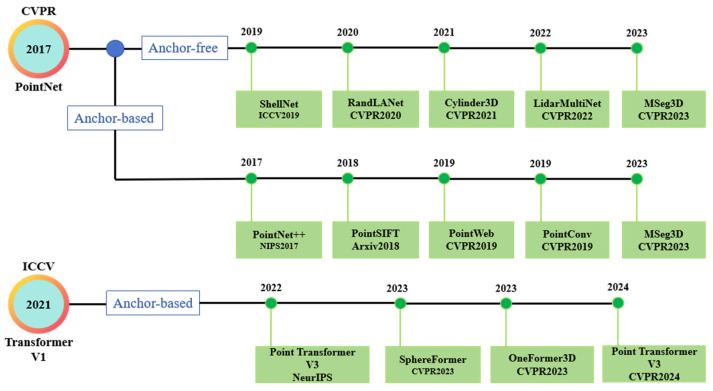
The development trend of point cloud algorithms.

Figure 1 illustrates the general development trends of point cloud processing algorithms, highlighting the transition from traditional geometry-based methods to learning-based approaches. It provides a conceptual overview of algorithmic evolution rather than a detailed performance comparison of specific models. In recent years, deep learning-based point cloud methods, such as PointNet and PointNet++, have been increasingly adopted in railway infrastructure applications, including component detection, structural segmentation, and damage identification. These methods demonstrate strong capability in learning discriminative features directly from irregular and unordered point cloud data, which is well suited to the complex geometric characteristics of railway scenes. However, their effectiveness still depends on factors such as data density, scene complexity, and computational resources.

## 2. Bibliometric Analysis

### 2.1. Research Data Sources

As an interdisciplinary research method, bibliometric analysis enables the exploration of the development context and cutting-edge trends within specific fields through quantitative analysis of a large number of documents. This approach assists researchers in intuitively and scientifically examining core problems and provides strong support for identifying and advancing research directions [19]. To ensure the comprehensiveness and authority of the data, the research data draws upon the Web of Science core database. Regarding the selection of keywords, the focus centers on two core concepts: “point cloud technology” and “transportation infrastructure”. The term “point cloud technology” encompasses related phrases such as “LiDAR point cloud”, “point cloud”, “point cloud processing”, “structured light point cloud”, and “3D scanning.” Similarly, “transportation infrastructure” includes terms like “transportation infrastructure”, “traffic infrastructure”, “railway”, “railway”, “subway”, “metro”, “free”, “expressway”, “highway”, “underpass”, “tunnel”, “bridge”, “viaduct”, and so on. The Boolean logic operator “AND” was employed to link these two concepts, thereby precisely defining the research topic. The time span for the data collection was set from the year 2000 to February 2025. After a rigorous deduplication and screening process, the literature retrieval settings are restricted to articles, review articles and conference papers, with a focus solely on English-language publications. This ensures the standardization and applicability of the data. The subject categories are carefully chosen to include transportation, transportation science technology, computer science, engineering, building technology, and other fields that are closely related to the research topic. This comprehensive categorization aims to capture the multi-dimensional research outcomes of point cloud technology in the field of transportation infrastructure. Ultimately, a total of 1805 eligible references were identified, including 748 in the field of bridges, 482 in tunnels, 341 in roads, and 234 in railways. These documents originate from 39 countries and regions, published across 428 journals, and involve the contributions of 1547 researchers, thereby reflecting the extensive scope and depth of research in this field. Given the relatively limited research in the railway sector, this review specifically focuses on this area.

Point cloud technology demonstrates significant value in railway infrastructure inspection, monitoring, and digitalization. Its advantages of non-contact, high-precision, and efficient operation enhance intelligent maintenance, with mobile measurement further expanding its applications. However, research in railways lags behind that in bridge and tunnel engineering, mainly due to challenges such as suppressing dynamic interference, meeting ultra-high precision requirements, processing massive point clouds in real time, and adapting to harsh environments. Current research focuses on AI-driven automated extraction, multi-source sensor fusion, and digital twin integration. Future development depends on optimizing hardware and software for railway conditions, promoting standardization, and improving cost-effectiveness to fully leverage its potential throughout the railway lifecycle, thereby advancing railway intelligence and modernization.

### 2.2. Literature Research Development Statistics

Vosviewer, a powerful bibliometric network construction and visualization tool, can efficiently process large volumes of information and present the analysis results in an intuitive graphical format. This functionality aids researchers in quickly discerning the underlying patterns and trends within the data [20]. Therefore, this study will export the selected references in plain text format and conduct an in-depth bibliometric statistical analysis using Vosviewer 1.6.20 software. Through this software, data visualization can be achieved across multiple dimensions, such as creating spatial distribution maps of literature, keyword co-occurrence maps, and literature co-citation networks. These visualizations provide strong support for subsequent in-depth analyses of research trends, hot issues, and development trajectory of point cloud technology in the railway sector.

**Figure 2:**
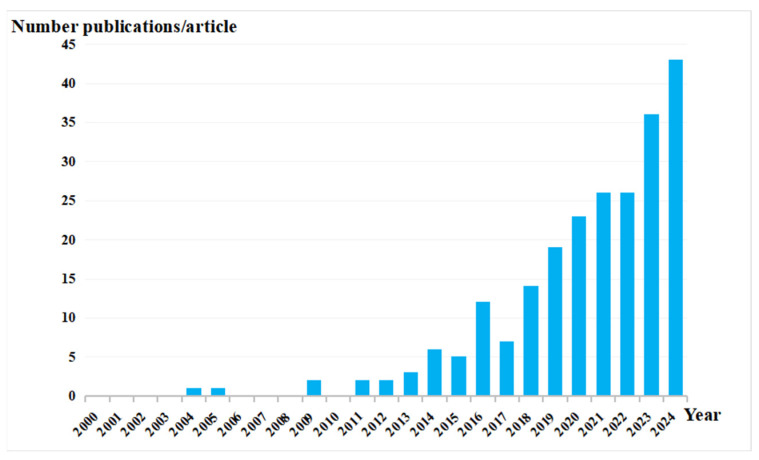
Point Cloud Enables Distribution of Literature Publication in the Railway Sector.

The annual number of published papers serves as a critical metric for assessing the development trajectory of scientific research. The temporal fluctuations in publication volume accurately reflect the evolving dynamics of knowledge within a particular field. Consequently, the volume of literature is one of the essential indicators for evaluating the extent of knowledge in a given area [21]. As shown in Figure 2, an examination of the changes in the number of published papers regarding point cloud technology in the railway sector from 2000 to 2024 reveals that the early relevant research was in its embryonic exploration stage, characterized by a limited number of publications, with fewer than five papers published annually across categories. However, with ongoing technological advancements and increasing demand for high-precision data in the transportation industry, the number of published papers has shown a consistent upward trend since 2018, particularly after 2022. For example, innovative research in areas such as railway track detection and disease monitoring of railway bridges and tunnels has emerged, which highlights a growing interest in this field and attracts the attention of an increasing number of researchers.

To explore the research contributions of various countries and regions in point cloud technology for railways, this study utilized Vosviewer software to examine the spatial dimension of literature publications. An in-depth analysis was conducted across 32 countries and regions, with a minimum threshold of one published document for visualization results. In this network, each item is a node, and the connections are links. The strength of these links depends on the distance between two nodes. A larger gap between two keywords or nodes signifies a lower correlation, whereas a smaller gap indicates a stronger correlation [22]. The total link strength represents the sum of link strengths associated with each node. In addition, the size of each node corresponds to the number of papers associated with the term, while various colors denote different years of research activity [23].

Bibliometric analysis indicates that the application research of point cloud technology in the railway sector has undergone a remarkable surge: it was in the embryonic stage before 2018, stepped into a phase of steady growth after 2018, and has presented an explosive momentum since 2022, particularly in the areas of track detection and bridge-tunnel monitoring. This trend underscores that the technology has become a research hotspot in railway intelligent operation and maintenance and is now in a phase of rapid development and in-depth application.

**Figure 3:**
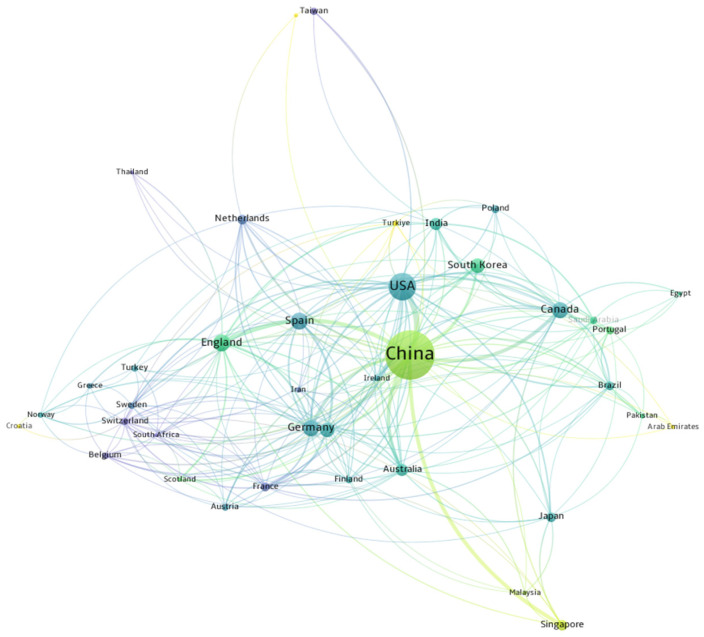
Spatial Distribution of Literature in Railway Enabled by Point Cloud.

Figure 3 illustrates a notable geographical imbalance in the literature published within the railway field, characterized by a significant concentration of publications from a limited number of countries. China, the United States, Spain, the United Kingdom, and Canada are the top five countries based on publication volume. China has achieved considerable research output in the railway field and has published a total of 108 papers published, which accounts for 46.15% of the overall publications in this field. However, the average citation count is relatively low, at only 9.64 citations per paper. In contrast, Spain has published 10 papers, accounting for 4.27%, while its average citation count is as high as 21. This indicates that the literature from Spain in this field has garnered significant attention and recognition in the academic community.

Research in the global railway sector exhibits distinct regional concentration, with five countries—China, the United States, Spain, the United Kingdom, and Canada—occupying a dominant position. Among them, China leads by an absolute margin in the number of published papers, reflecting its strength in large-scale research capacity. Nevertheless, Spain boasts the highest average number of citations per paper, underscoring the academic influence and international attention garnered by its research outcomes. This pattern of quantitative and qualitative differentiation indicates that China drives the expansion of research volume, while Spain has forged unique advantages in the depth of innovation and research quality.

### 2.3. Literature Coupling and Keyword Co-Occurrence Network Analysis

**Figure 4:**
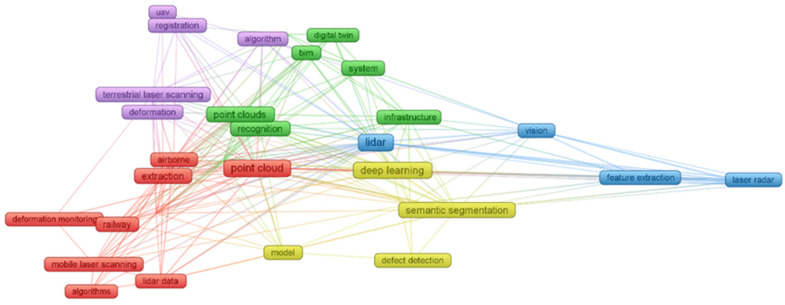
High-frequency keywords co-occur on the web.

During the bibliometric data collation, it was found that high-frequency keywords including “point cloud”, “lidar”, “deep learning”, “extraction” and “classification” form the core vocabulary system of this research field. For further cluster analysis, this paper focuses on the following five categories of research hotspots for discussion. The application of point cloud technology in the railway field, such as railway deformation monitoring and point cloud extraction via airborne and mobile laser scanning, which is marked in red in the figure. Point cloud data processing and analysis technologies, including the optimization and innovation of key algorithms such as data denoising, registration and segmentation, so as to improve the efficiency and accuracy of data processing, which is marked in yellow in the figure. The integration of point cloud technology with other cutting-edge technologies, such as its combination with artificial intelligence (AI) and building information modeling (BIM), to realize the intelligent management and full-life-cycle maintenance of transportation infrastructure, which is marked in green in the figure. Point cloud-related acquisition technologies, including terrestrial laser scanning and unmanned aerial vehicles (UAVs), which is marked in purple in the figure. Point cloud and lidar-related technologies, including lidar visual applications and feature extraction, which is marked in blue in Figure 4.

Based on the current bibliometric analysis, the future development of point cloud technology in the railway inspection field will accelerate toward the direction of multi-technology integration. On the one hand, with the continuous advancement of artificial intelligence technology, the automated processing and intelligent analysis of point cloud data will become a research priority, enabling more efficient and accurate inspection and evaluation. On the other hand, in the full life cycle management of infrastructure, the in-depth integration of point cloud technology with the Internet of Things (IoT), big data, and other technologies will foster a more sophisticated intelligent management system. Meanwhile, in response to the demands for large-scale inspection and more refined damage management, research on the application of point cloud technology in the railway digitalization field will also emerge as a new research hotspot.

**Figure 5:**
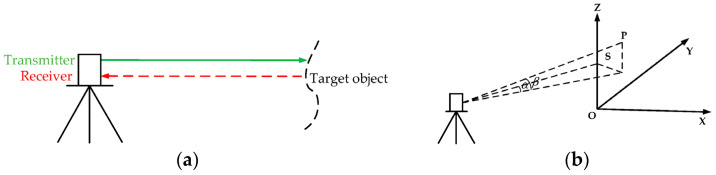
TLS principle. (**a**) Pulsed laser ranging, green for transmit and red for receive; (**b**) Geometric relationship [29].

With the continuous advancement of science and technology, point cloud technology has become increasingly prevalent and deeply integrated into the railway field, as it offers unique advantages that facilitate new opportunities and transformations in railway engineering. 3D point cloud data can be obtained from various sources, including laser scanning, 3D reconstruction of two-dimensional (2D) images, and video recordings [24]. In addition, depth maps captured by the depth camera can also be converted into point cloud data. Unlike 2D images, each pixel in a depth map possesses four attributes: red (R), green (G), blue (B), and depth (D) [25]. This capability facilitates a more accurate reconstruction of 3D information about objects. Among the various methods for acquiring 3D features, laser scanners are widely recognized for their precision, efficiency, and high-resolution data acquisition capabilities. These systems scanners can generate accurate 3D representations of objects [26]. LiDAR systems are categorized based on their platforms into airborne lidar scanning (ALS),Terrestrial Lidar Scanning (TLS), mobile lidar scanning (MLS), and unmanned lidar scanning (ULS) [4], where the principle of TLS is shown in Figure 5. Each type of laser scanner varies in resolution and spatial coverage. While laser scanners are less flexible and more costly than unmanned aerial vehicles (UAVs), they offer significantly higher accuracy in data acquisition and exert less environmental impact [27,28].

## 3. Track Infrastructure Inspection

### 3.1. Track State Detection

Building upon the fundamentals of point cloud acquisition and processing, recent studies have increasingly focused on extracting precise geometric information from railway infrastructure. Railway facility monitoring is of great significance to ensuring the safety of railway systems. LiDAR is gradually replacing traditional manual inspection and track inspection vehicle-based methods, emerging as a powerful tool for railway safety maintenance, and the point cloud data it captures can accurately delineate the geometric structures of objects in the railway environment [30]. Karunathilake, A. et al. [31] processed long-range laser scanning point cloud data by iteratively applying the vertical slicing technique, achieving a 100% accuracy rate in railway structure inspection while improving the performance of ground control (GC) point extraction in railway structure monitoring. F. Han et al. [32] proposed a method combining spatial distance threshold setting, principal component analysis calculation, and linear discriminant analysis; the extracted tracks reached over 94% in both completeness and accuracy. The research team [33] developed an algorithm that performs linear fitting on candidate points via the RANSAC algorithm to identify railway facility points, ultimately generating a continuous and high-quality 3D model for all railway tracks. Jianjie Wu et al. [34] proposed a hybrid segmentation algorithm integrating the augmented region-growing technique and improved alpha-shape method, which extracts track features from cluttered scene data using point cloud data collected by unmanned aerial vehicles (UAVs). Burdziakowski, P. et al. [35] put forward a novel framework for defining railway track axes in 2022, based on point cloud data acquired by UAVs through specific filtering and extraction processes; this framework is applicable to technical track monitoring, geographic information mapping, and cartographic tasks. In addition, Yihao Ren [36] developed an automatic track extraction method suitable for low-density datasets in 2024, which is not restricted by sensor attributes or global features and achieves an accuracy rate of 99.7%.

The fasteners in the railway system are critical for the safe operation of tracks. Existing methods for evaluating tightness often suffer from limitations such as low accuracy and efficiency, as well as susceptibility to interfere and misjudgment. To address the deformation and elastic change characteristics of fasteners, Wang et al. [37] proposed a high-precision surface structured light technology based on centerline projection distance, along with a neural network-based fastening regression method. The root mean square error (RMSE) deviation between the regression value generated by the algorithm and the actual measurement is only 0.2196 mm. This represents a significant improvement over other methods, achieving effective regression. The intricate shapes of railway fasteners can easily lead to spatial damage defects, while the data imbalance in real-world scenarios hampers the effectiveness of deep learning models in detecting and quantifying damage. Therefore, Wang et al. [37] proposed an innovative railway fastener point cloud analysis method based on deep learning and constructed a dataset comprising 120 real and virtual damaged fasteners. The average intersection-over-union ratio for the point cloud segmentation task reached 99.35%, which significantly improved the efficiency of railway safety detection. Traditional methods for detecting railway fastener sealing predominantly rely on manual annotation tools such as labelme, which are error-prone, labor-intensive, and costly. Additionally, monocular depth estimation and instance segmentation calculation can be complex, which is not conducive to real-time implementation, particularly on the resource constrained platform. Qiu et al. [38] introduced a three-phase solution using a multimodal geometric automatic encoder (MGAE), which integrates point cloud with monocular depth-guided multimodal data. This solution extracts uses a mixed autoencoder to extract high-quality features in an unsupervised manner and integrates multiple data types and fusion blocks to enhance performance, thereby achieving accurate sealing estimation. Given the substantial number of railway fasteners that require regular inspection, traditional manual inspection methods are typically laborious and inefficient. Consequently, Cui et al. [39] developed a real-time inspection system based on point cloud in-depth learning. This system acquires and segments point clouds using structured light sensors to avoid debris interference. A semantic segmentation dataset was created and tested for Pointnet++ deployment. Field tests demonstrated that the system achieved high accuracy and efficiency in ballasted railway inspection.

Deep learning is endowed with the capability of automatically extracting features from data, which has vigorously advanced the development of the computer vision field. At present, it has been widely applied to numerous tasks such as object classification, localization, and semantic segmentation. A great number of researchers have leveraged this method to process the collected point cloud data, thereby efficiently achieving component localization and fault identification. Soilán et al. [40] proposed a fully automated method that starts with 3D point cloud data of railway infrastructure and sequentially executes heuristic-based point cloud processing steps to accurately extract and delineate the position and geometric shape of steel rails, with the ability to export IFC-compliant files describing rail positions. Hu et al. [41] introduced a rail extraction algorithm that enables real-time processing of mobile laser scanning data by utilizing a coarse-to-fine hierarchical approach for rail feature extraction and a newly defined track descriptor. This algorithm achieved high extraction accuracy and effective real-time performance. Liu et al. [42] presented a point cloud reconstruction method for a mobile laser scanning system that utilizes trajectory filtering. With the help of DL to identify railway feature points and correct odometer data, the multi-sensor integrated mobile detection system is crucial for advancing intelligent railway detection. Narazaki et al. [43] proposed a vision-based autonomous navigation strategy for UAVs, which is employed for rapid inspection of reinforced concrete railway viaducts after earthquake. It enables the automatic identification and localization of key structural components. This method achieves stable and complete detection of chromatographic columns with centimeter-level accuracy. Railway track surface extraction from point clouds is a critical technology for railway inspection; however, variability in the quantity, density, and scene of point cloud data presents significant challenges. B. Yang and L. Fang [44] proposed a method for the automatic detection of railway tracks from mobile laser scanning (MLS) point clouds. Both the geometric and intensity data of railway tracks were utilized to extract track points and establish track models.

Processing large and dense point cloud data sets often entails high computational demands and extended execution time. To address these challenges, Cserép et al. [45] implemented and conducted a comparative analysis of railway fragmentation and object segmentation algorithms. They focused on robustness and efficiency while prioritizing automation and reducing preconditions, such as the spatial relationship between railway tracks and overhead contact lines. This approach allows for the easy parallelization of processing across large railway sections. Kononen et al. [46] presented a real-time algorithm for double-track railway configurations that utilizes solely geometric data. This algorithm is capable of extracting the top centerlines of up to seven parallel tracks in a single measurement. It can achieve accuracy levels between 0.3 cm and 0.8 cm on non-intersecting guide rails, which represents a 55% to 85% improvement over previous techniques. Grandio et al. [47] introduced a multimodal DL-based approach for panoramic segmentation of railway infrastructure. This method employs image rasterization of point clouds for preliminary segmentation, which effectively discards over 80% of irrelevant points. This significantly reduces computational load for processing the remaining point clouds and enables the rapid handling of dense point clouds. An automatic and configuration-independent coarse-to-fine extraction method was proposed for low-density LiDAR data [48].

**Figure 6:**
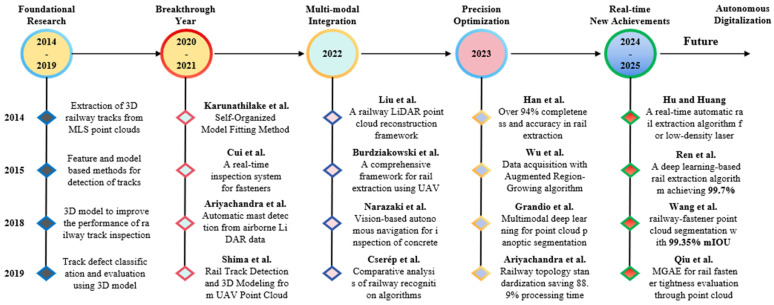
The field of railway inspection is a major development trend.

In the realm of railway operations and upkeep, precise geometric information model reconstruction is pivotal for ensuring efficient operations and significantly enhancing overall work efficiency, as in Figure 6. Mahmood et al. [49] achieved seamless integration with BIM through standardized derivation of geometric parameters from point cloud data, which enabled the calculate of parameters for station points and the generation of associated data for new station points along the track. This advancement improves defect localization capabilities and contributes to the efficient management of railway track lifecycle. Geometric information modeling based on point cloud data serves as the basis for creating DTs of railway infrastructure; however, existing methods often struggle to balance accuracy, labor costs, and large-scale demand. Ariyachandra et al. [50] used highly standardized railway topology to automatically generate geometric information model of railway track structure, which can reduce generation time by 88.9% without manual intervention. When automatically creating geometric DTs from railway point cloud data, a primary challenge is detecting masts from airborne LiDAR data. Ariyachandra et al. [51] utilized the high supervision and standardization characteristics of the railways to develop countermeasures that successfully achieved a total physical examination rate of 94%, which set a precedent for the automatic detection of masts from airborne LiDAR data.

Currently, the point cloud information concerning track infrastructure primarily focuses on detecting track geometry, foreign objects between tracks, and significant diseases and damages. The advancement of high-precision point cloud information acquisition equipment enables the extraction of minor defects such as rail surface diseases and bolt fastener cracks. In the future, the integration of point cloud image fusion data with machine vision is expected to reveal substantial potential for the intelligent operation and maintenance of rail infrastructure. This will drive the industry toward more refined and intelligent development.

### 3.2. Catenary Detection

**Figure 7:**
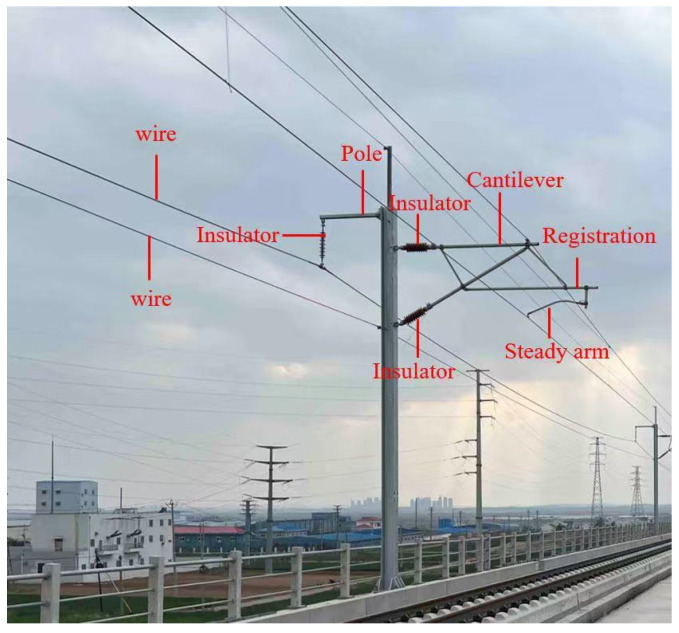
OCS structure.

High-speed railways constitute an essential part of transportation infrastructure. However, the expansion of operating mileage poses significant challenges to ensuring safe, reliable, and efficient operations. As shown in Figure 7, the contact network (OCS) that supplies power to high-speed railroads consists of several complex components, which are prone to deformation, loosening or even falling off due to natural factors such as ice, snow and wind. These issues can lead to changes in geometric dimensions and potential accidents. Consequently, regular identification and inspection of OCS components are essential to ensure the safe functioning and upkeep of high-speed railways, which aids in the reduction of accidents associated with these systems [12]. Nonetheless, the variety of OCS components complicates the identification and detection processes. Traditionally, manual methods relying on professional technicians and handheld devices have proven inefficient and imprecise [52]. They are not able to adapt to the ongoing increase in operating mileage and hours of modern high-speed rail systems. In this context, automatic patrol inspections have emerged as a standard practice, and the 3D information contained within point cloud data is crucial for measuring geometric parameters during catenary inspection. Therefore, effectively identifying OCS components from point cloud data collected by detection equipment is crucial for advancing the automation of parameter measurement.

As technology progresses, LiDAR technology has been increasingly applied to railway infrastructure detection. It can collect 3D point clouds containing both environmental geometry and radiation data using mobile laser scanning system. These point clouds enable the automatic identification of specific OCS components, measurement of specific parameters, and detection of any faults. Han et al. [53] introduced the state detection method based on the concave and convex characteristics of 3D point cloud data from contact wire systems (CSCs), which addressed the inefficiencies associated with manual measurements. Geng et al. [54] proposed a measurement framework based on UAV LiDAR technology to achieve high-precision and efficiency measurements of static parameters for contact wires along the entire length of high-speed railways. This framework successfully controlled the measurement error within 9 mm. This advancement represents a significant innovation in inspection methodologies for high-speed railways. Mahtani et al. [55] proposed an innovative method that integrates infrastructure surveying and mapping system with point cloud analysis. Subsequently, they applied it to railway track and catenary sensing, which provided new insights for ensuring the operational safety of autonomous trains. Sánchez Rodríguez [56] developed a method for the automatic assessment of deflection in gap gauges and air contact lines within railway tunnels. This diagram depicts a point cloud processing pipeline where the input point cloud undergoes parallel feature extraction and a three-stage attention-based refinement (with dropout regularization in the second stage). The fused features are enhanced via a residual connection, then split into two branches: one branch applies log softmax, reshaping, transposition, and convolution with dropout for feedback refinement, while the other is directly reshaped to produce the final result. These studies lay a foundation for further exploration of OCS detection technology.

**Figure 8:**
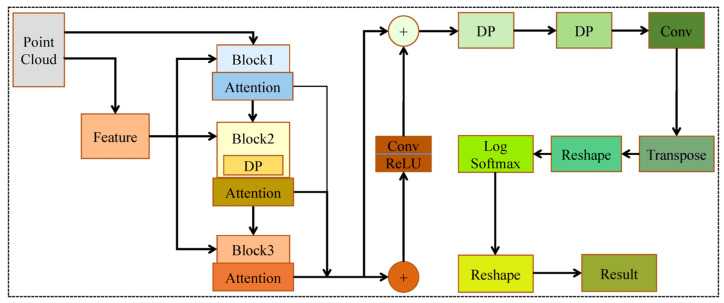
RobotNet model [57].

Despite these advancements, using LiDAR for OCS detection encounters challenges related to point cloud data segmentation and component recognition. The numerous OCS components and the complexity of railway environments complicate identification efforts. However, DL techniques, which enable automatic and efficient extraction of high-dimensional features, have led to the development of various models for point cloud recognition. For example, Tu et al. [57] introduced a lightweight model, Robotnet, as shown in Figure 8, which utilizes point convolution and attention modules for point cloud identification, and employed compilation tools to optimize its recognition speed on embedded devices. In addition, they developed a visualization software capable of displaying extensive point clouds and details of OC components. Experimental results demonstrated that this model achieves enhanced accuracy and efficiency in OC component recognition. Chen et al. [58] proposed a new DL-based method that incorporates an iterative point division algorithm and a spatial fusion network module for multi-scale local feature extraction. Additionally, they performed semantic segmentation on point clouds collected using a portable 2D laser scanner. The experimental outcomes confirmed the effectiveness of this method in multi-target identification. Liu et al. [59] presented a lightweight neural network EffNet, which consists of extraction, attention, and enhancement components. By optimizing the process of feature extraction and enhancement, this network reduced computational and model complexity while achieving higher average accuracy in OCS recognition. Wei et al. [60] proposed an innovative intelligent scheme integrating deep learning and image processing technology—PDDNet—for the online condition monitoring of pantograph slides. This scheme can not only detect surface defects and accurately identify four types of defects but also demonstrate high accuracy in wear depth estimation. However, existing deep learning-based recognition methods generally suffer from problems such as high computational complexity and room for accuracy improvement. To effectively utilize texture information, some methods opt to perform recognition and detection tasks for the overhead catenary system (OCS) by means of images.

Huang et al. [61] proposed a progressive detection strategy called Joint horizontal-vertical enhancement and tracking (JHVET), which is designed to accurately detect geometric parameters of pantograph-catenary contact points from infrared images in complex backgrounds. This method achieved a detection accuracy of 98.23%, an average pixel error as low as 0.523 pixels, and a detection rate exceeding 108 fps. This method demonstrated excellent performance and provided a novel solution for research in this field. Although researchers frequently employ DL to analyze 2D images for predicting the status of OCS components, this method is inherently limited in its ability to accurately measure the geometric parameters of OCS. In contrast, point cloud-based detection can generate detailed metrics for each OCS component, significantly improving precision in OCS detection efforts [11].

## 4. Railway Environmental Monitoring

### 4.1. Landform and Geological Monitoring

Beyond infrastructure condition assessment, point cloud technologies have also been increasingly employed for foreign object and obstacle detection in railway environments. The railway plays a pivotal role in the modern transportation system, and its safe and stable operation is directly related to the national economy and public welfare. Topography and geological conditions are fundamental to railway construction and operation. They affect every phase, from the initial line planning and foundation construction to ongoing maintenance efforts that address natural and human interference during operations. Therefore, an in-depth study of topography and geological monitoring is critically significant for the railways, as it not only enhances the efficiency and safety of current railway construction activities but also has lasting implications for the sustainable development of the railway system.

**Figure 9:**
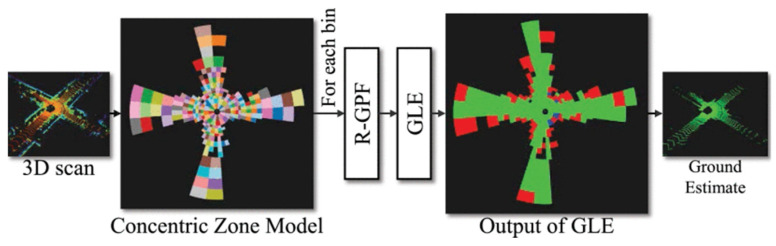
Patchwork Overview [62].

In addressing the complex terrain and landform involved in railway construction and operations, numerous research findings have provided effective methods for addressing practical challenges. For example, uneven ground conditions complicate the navigation of ground mobile platforms and the recognition of adjacent objects. Lim et al. [62] proposed a novel ground segmentation method called patchwork, as shown in Figure 9, which encodes the point cloud into a representation based on the concentric zone model. This method estimates the grounding for each segment through region -wise ground plane fitting and employs ground likelihood estimation to minimize false positives. Experimental results using the Semantickitti and rough terrain datasets indicated that this approach significantly outperformed existing advanced methods in terms of both accuracy and speed, achieving an operating frequency exceeding 40 Hz. In carbonate rock mass fields, landslides and rockfalls often arise from the movement of discrete blocks and the presence of caves. Loiotine and Lidia [13] integrated traditional field surveys with remote sensing technologies, including TLS, LiDAR, and UAVs, to process point cloud data through case studies. They extracted rock mass discontinuity information, evaluated the advantages and disadvantages of various technologies, and constructed accurate 3D models. By utilizing kinematic analysis to identify potential instability factors and applying these results to numerical calculations, they assessed the response of rock mass to gravitational forces. In addition, terrain change detection is crucial for identifying and monitoring geological hazards such as landslides, which contributes to risk-based decision-making. Expanding this capability from a site scale to a regional scale facilitates proactive asset management and enhances the resilience of infrastructure.

Sinkhole settlements have caused significant damage to traffic infrastructure, with the occurrence of such events rapidly increasing in karst regions globally. Quantitative characterization of deformation is essential for devising effective mitigation measures and preventing potential accidents. However, research on active sinkhole monitoring with high-precision and high-resolution techniques remains limited. To address this gap, Sevil et al. [63] proposed a sinkhole monitoring method that combines high-precision leveling and high-resolution TLS. This integrated approach allows for the accurate depiction of the spatio-temporal settlement patterns and boundary positions of the area affected by land deformation. Luo et al. [64] utilized TLS, global navigation satellite system (GNSS), and UAV to study surface deformation evolution in two frost mounds located in QTEC. They revealed deformation characteristics of slopes across different periods and highlighted the critical roles of both TLS and hydrothermal measurement technologies in assessing slope hazards along QTEC and informing the design and construction of high-speed railways. Although directly applying 3D algorithms to the original point clouds can enhance accuracy in change detection, this approach significantly increases computational demands. Weidner and Luke [14] introduced a new ICP-M3C2 workflow that is based on GPU processing, which allows point cloud data segments to be automatically queued for GPU processing while the CPU manages data in parallel. This method is reported to be 54 times faster than the CPU-based version and had been applied to six regional-scale landslide identification and monitoring cases, including the interruption of pipelines, highways, and railway corridors resulting from landslides. In 2021 and 2022, this method was utilized to process linear change detection over more than 17,500 km of railway.

**Figure 10:**
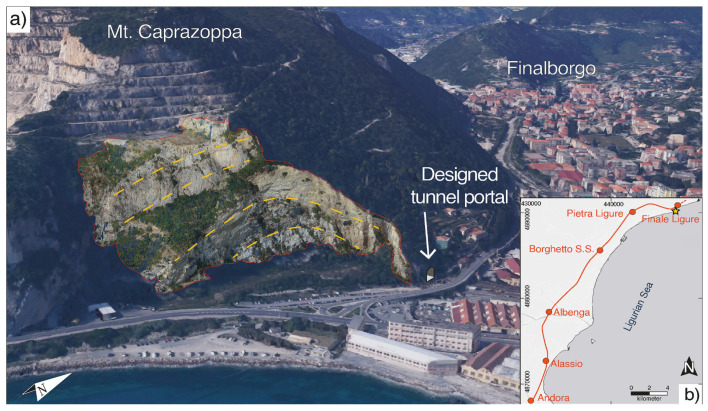
(**a**) DOM perspective view; red line represents RTK UAV photogrammetric survey area, yellow line represents local fold geometry (**b**) Path of the new rail line from Finale Ligure Marina to Andora; red circles represent train stops, yellow stars represent DOM locations [66].

The maintenance of railway facilities is crucial for ensuring the reliable operation of railways. Reverse Engineering (RE) can enhance maintenance efficiency within this context. However, creating BIM often requires numerous manual tasks based on point cloud data, which are labor-intensive and time-consuming. Park [65] identified representative parameters of straight-line single-segment ballasted track using point cloud data and compared this scanning data with BIM to assess damage types. A ballast test bench was utilized to gather point cloud data, which was used to develop a BIM model. Moreover, the practicality of developing a railway upkeep BIM using characteristic three-dimensional target identification metrics in the context of the ballasted track was evaluated. Perozzo, M. [66] used digital outcrop models (DOMs) acquired by UAVs to quickly and quantitatively characterize rock mass structures in complex geological environments, enabling more accurate and targeted kinematic assessments of rock slopes, as shown in Figure 10. This study takes the Finale Ligure area as an example and specifically analyzes the track replacement project from Finale Ligure Marina to Andora station. This approach underscores the significance of integrating digital workflow with field characterization to effectively utilize large datasets and automated programs, which allowed for the quantitative definition of potential unstable areas through rapid analysis. These studies offer innovative ideas and methodologies for addressing topographical and geological challenges in railway construction, operation, and maintenance. These studies establish a strong foundation for further investigation and provide significant theoretical and technical support for railway industry development.

### 4.2. Foreign Object Detection

**Figure 11:**
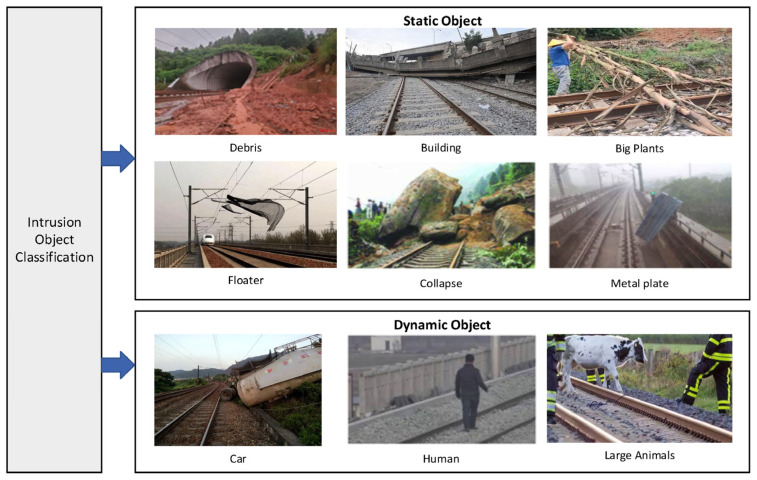
Classification of intrusion targets [67].

Lately, concerns regarding the safety conditions surrounding high-speed rail systems have grown significantly. Adverse weather conditions, along with the intrusion of individuals or obstacles into the surrounding area, frequently lead to serious accidents, including derailment and parking. Common intrusion objects are categorized as shown in Figure 11. Given the limitations of single sensors in meeting the comprehensive monitoring demands in complex application scenarios, research on multi-sensor fusion detection technology has emerged as a significant topic. This approach leverages the combined strengths of various sensor modalities, including images and point cloud data, to enhance perimeter intrusion detection in high-speed railway systems [67]. Han et al. [68] successfully achieved real-time detection of small target obstacles using a 16-ray 3D LiDAR system for data acquisition and point cloud imaging. Qu et al. [69] developed an obstacle detection method based on LiDAR to mitigate accidents caused by obstacles encroaching upon the railway corridor. Their methodology involved preprocessing the railway scene point cloud to delineate the basic area containing rail, followed by the application of the RANSAC algorithm for fitting the subgrade plane and defining the detection area. To address the limitations of traditional Euclidean clustering algorithms, they introduced an adaptive distance threshold method for obstacle classification. Experimental results demonstrated that the improved algorithm is superior to the traditional method in terms of calculation time and segmentation accuracy, successfully detecting railway obstacles.

**Figure 12:**
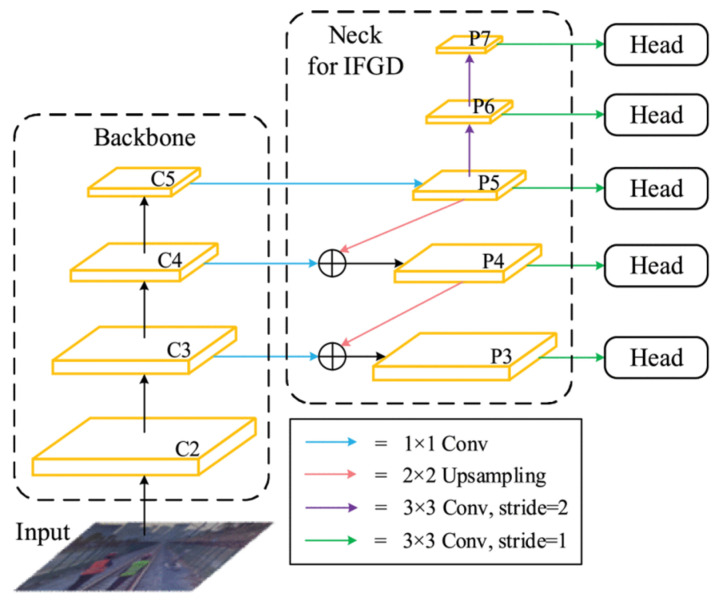
RFA-Net Network Structure [74].

Similarly, the point cloud obstacle detection algorithms have garnered considerable attention. To mitigate ground disturbance, Li et al. [70] proposed an obstacle detection algorithm that combines 3D LiDAR and two axis rotating platform, which can identify obstacles measuring 10 cm in height. However, the operational range of this technology is limited, which underscores the urgency for developing robust LiDAR equipment and algorithms capable of detecting small obstacles within specific ranges. Franke et al. [71] introduced a method for generating synthetic images of long-distance obstacles on or near the rail, which effectively improved the detection capabilities for remote objects. Perić et al. [72] developed a system to detect railway obstacles and track intrusions. Its core decision support system (DSS) utilizes machine learning algorithms to analyze sensor images to support decision-making. Rampaya et al. [73] demonstrated that both SSD Mobile networks and Faster RCNN perform well in detecting railway obstacles. This is an object detection system where an input image is processed by a backbone network to extract multi-scale features, which are then merged and refined by the IFGD neck module, with each final feature map sent to a detection head to identify objects of different sizes. The former achieved an accuracy of 96.75%, while the latter reached 84.75%, even under different lighting conditions. Qin et al. [74] developed a robust function aware network (RFA-Net) that significantly enhanced the efficiency of feature extraction without increasing the computational burden, as shown in Figure 12 The lightweight RFA-Net model achieved a detection accuracy of 92.7% mAP. It can reliably detect obstacles in complex rail transit environments while exhibiting excellent generalization capabilities. Wang et al. [75] combined a progressive clustering approach and lightweight segmentation methods to overcome challenges posed by unknown or unforeseen obstacles in railway transportation.

In the field of semantic segmentation for railway point clouds, Feng et al. [76] developed a DL-based trajectory recognition and detection system. This system employs a convolutional neural network for extracting rail features and implements the Mask R-CNN instance segmentation algorithm. It can not only accurately identify rails but also reasonably delineate hazardous areas, demonstrating strong anti-interference capabilities and high levels of accuracy. Jiang et al. [77] enhanced the understanding of railway point cloud semantic segmentation by proposing a DL-based framework called RailSeg, which encompasses point cloud subsampling, combined local and global feature learning, spatial context fusion, and semantic refinement, thereby facilitating advancements in 3D semantic segmentation, digital railway, and intelligent transportation. Manier et al. [78] innovatively proposed a DL-based method for 3D point cloud semantic segmentation within real-world LiDAR railway scenes. This approach uses spatial local point cloud transformation for convolution learning, which not only enhances the adaptability to point clouds of varying densities but also ensures that measurement information and descriptive capability are preserved.

**Figure 13:**
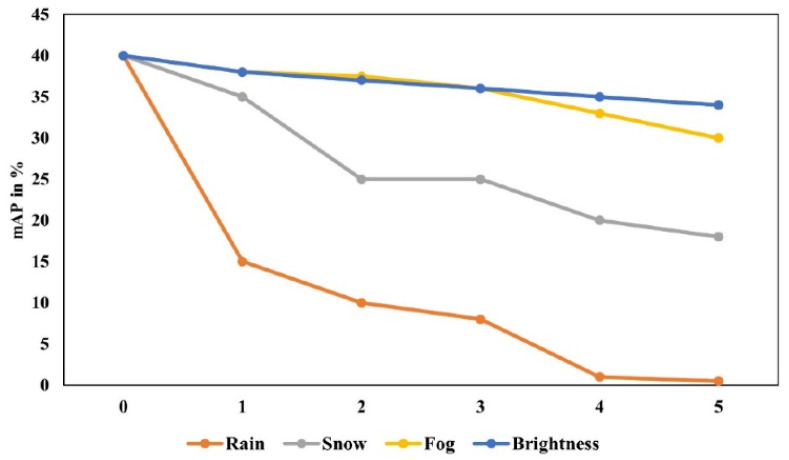
Camera Detection Accuracy under Weather Interference [80].

Nevertheless, adverse weather conditions significantly hinder detection performance. Wang et al. [79] pointed out that the interference from rain, snow, and fog can reduce the accuracy of camera-based detection by a minimum of 31.1% and up to 60.4%. The severity of weather conditions directly correlates with decreased detection effectiveness, as shown in Figure 11. Appiah et al. [80] developed an enhanced approach utilizing YOLOV7 to improve the detection performance in inclement weather. Despite some improvements, the detection accuracy remains below 80%. Shi et al. [81] introduced SMIFormer, a multi-view feature fusion network framework grounded in four-dimensional (4D) radar single-mode input. This framework decomposes 3D point cloud environment into three distinct but related viewpoints: bird’s eye view (BEV), front view (FV), and side view (SV). It can better model the overall 3D scene and effectively address the limitations of single-view feature representation in extremely sparse point cloud scenarios. To improve the point cloud 3D semantic segmentation (3DSS) for track obstacle detection in complex weather, Wen et al. [82] introduced a multimodal comparative learning strategy, DHT-CL, which integrates visual and LiDAR-based data sources, tailored for challenging weather environments and the identification of obstacles. As shown in Figure 13, it presents the intensity distribution under different weather conditions like sunny, raining, and after snow, highlighting the challenges and scenarios that DHT-CL aims to address. This method eliminates the need for input images during reasoning. It enhances detection precision under sunny conditions and lowers the incidence of false alarms during rain and snow. When compared to LiDAR and cameras, 4D radar exhibits superior performance under adverse weather conditions. Wang et al. [83] proposed an obstacle detection method based on 4D radar within train running contexts, which dynamically adjusts the detection range of obstacles using the DBSCAN algorithm.

Point cloud technology holds significant promise in the field of railway environmental monitoring and foreign object intrusion detection. By geometrically and parametrically analyzing the railway environment and foreign object intrusions, this technology transforms complex scenes and objects into quantifiable data. This transformation enables researchers to acquire relevant information with greater accuracy, thereby greatly advancing research in this field and providing strong support for ensuring railway system reliability and stability. The successful application of point cloud-based modeling in track geometry has encouraged further exploration of its use in railway structural inspection and condition assessment.

## 5. Health Monitoring and Detection of Railway Bridges and Tunnels

### 5.1. Bridge Structure

As the railway industry thrives, both the maintenance and upgrading of existing lines and the planning and construction of new ones have focused on the technical research of railway bridges, which are vital pieces of infrastructure. This includes the key areas such as detection, monitoring, and digital modeling. TLS measurement demonstrates excellent vertical displacement recognition capabilities. Gawronek et al. [84] reported that the TLS measurement results are consistent with the traditional measurement height, achieving a minimum accuracy of ±1 mm. Moreover, modern 3D scanning technology has introduced innovations in the processes of replacing railway bridge sleepers. Najafi et al. [85] introduced a semi-automatic geometric feature extraction framework that significantly enhances the accuracy and efficiency of sleeper replacement procedures. The continuous emergence and development of new computer vision methods are anticipated to mitigate the high costs associated with manual bridge measurements. As railway traffic volumes rise, bridge maintenance has become increasingly critical. Poku-Agyemang et al. [86] introduced an innovative workflow for bridge digitization from 2D plans using existing measurement methods such as laser scanning and photogrammetry. This process involves corner detection through image processing and the creation of scaled 3D models using point cloud reconstruction and fusion technologies. Additionally, point cloud data serve as a valuable complement to laser scanning and camera data.

In the field of point cloud data processing and scene segmentation, Qiu et al. [87] employed a fraction-based noise reduction algorithm to process low-quality point clouds obtained by UAVs. They then utilized an improved DGCNN to segment common infrastructure present in the railway bridge point clouds. This operation successfully extracted the key features from the low-quality point cloud and achieved accurate digital representations of railway bridge scenes. Shen et al. [88] proposed a method that considers the geometric and topological constraints of bridge components by employing a coarse-to-fine and top-down strategy. This method can automatically segment key components of long-span railway cable-stayed bridges, such as piers and stay cables, from point clouds acquired via UAV LiDAR. The dataset demonstrated an average accuracy exceeding 96% at the point level. Furthermore, Chen et al. [89] introduced a process enabling the automated segmentation and reconstruction of railway structures based solely on point cloud data, without dependence on intensity or trajectory inputs.

**Figure 14:**
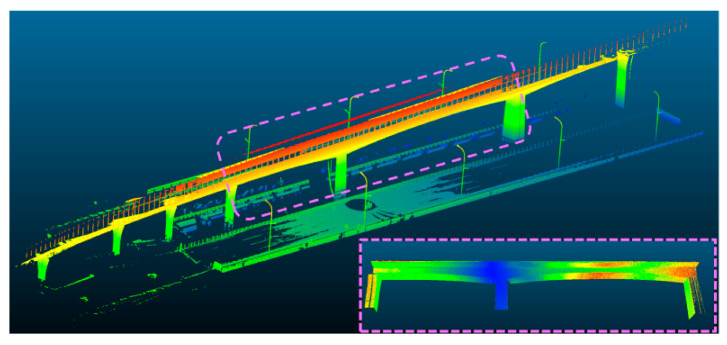
Point cloud model of the test bridge [91].

The analysis of bridge dynamic behavior is crucial for ensuring the safe operation of railway bridges. With the large-scale expansion of China’s high-speed railway network, there is an urgent need for reliable and efficient detection methods applicable to multi-track and long-span railway bridges. Huang et al. [90] used an IBIS-S ground microwave radar interferometer in conjunction with laser radar point cloud to monitor vibrations of the Nanjing Daguan high-speed railway bridge (NDHRB), which is flanked by subway tracks. This approach demonstrated significant advantages in spatial resolution (0.5 m) and temporal resolution (50 Hz–200 Hz), which enables engineers to accurately measure the maximum displacement of the bridge due to train movements and effectively monitor bridge dynamics from a horizontal perspective, thus distinguishing the impacts of varying traffic conditions. Wu et al. [15] employed a BP neural network to perform high-precision 3D modeling on point cloud data obtained from a 3D laser scanner for monitoring the deformation of a maglev train bridge. Their experimental analysis confirmed observable deformation effects on the bridge when trains passed over it. Zhou, Yin [91] proposed a TLS-based deformation pattern monitoring method for bridges by developing a SWSF deformation pattern calculation method combining sliding window and surface fitting through the point cloud model in Figure 14. Compared with the total station method, there is no need to pre-set reflective markers, which not only enhances the deformation monitoring resolution from millimeter to sub-millimeter scale, but also converts discrete measurement point data into continuous spatial deformation profiles. Lee et al. [16] constructed a displacement extraction framework that is based on geometric principles, which can automatically retrieve measured values of key infrastructure in sequence. This framework constructs a comprehensive geometric profile of the bearing by projecting the curvature of the point cloud onto the best-fit circle, which allows for the extraction of required displacement measurements.

In the context of diversified and integrated bridge inspection methods, the fusion of multi-source data has emerged as a significant trend. For instance, Olaszek et al. [92] combined a scanning total station (STS) with an unmanned aerial vehicle (UAV) to verify bridge dimensions. This approach leverages the high direct measurement accuracy and geometric analysis capabilities of the STS, while the UAV ensures efficient coverage, achieving over 90% compliance with reference dimensions. This synergy enhances both operational safety and cost-effectiveness. To address limitations inherent to single data sources—such as incomplete imagery of near-ground structures, difficulties in capturing bridge deck data, and the lack of texture in point clouds—Li et al. [93] successfully generated an accurate, comprehensive, and realistic 3D bridge model by fusing UAV imagery with terrestrial laser scanning (TLS) point clouds, providing a key technical reference for 3D reconstruction.

**Figure 15:**
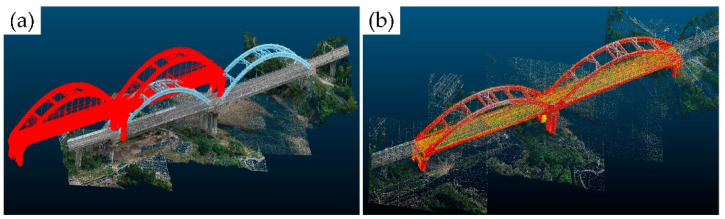
Fusion of TLS point cloud and UAV point cloud (**a**) before fusion; (**b**) after fusion [93].

**Figure 16:**
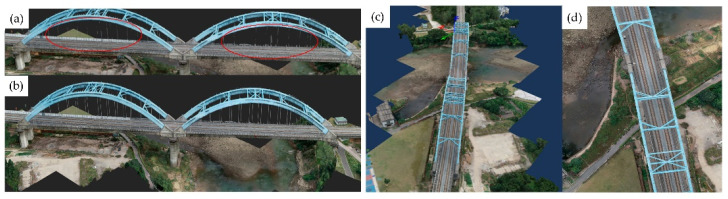
Fused 3D real-world models: (**a**) 3D real-world modeling by a single drone; (**b**) Com-bination of drone and terrestrial laser scanning for 3D real-world modeling; (**c**,**d**) Partial textured results of the integrated modeling [93].

Figure 15 and Figure 16 conceptually illustrates a typical multi-source point cloud fusion process (e.g., TLS and UAV) within a railway scenario, demonstrating its general feasibility. In practice, such integration is commonly achieved through coordinate system unification using control points or GNSS/IMU assistance, followed by point cloud registration via methods like Iterative Closest Point (ICP) or feature-based alignment. It should be noted that fusion accuracy varies depending on sensor configurations, acquisition conditions, and application requirements. A detailed quantitative comparison of methodologies will be a focus of future specialized research. Lee et al. [94] proposed a non-target-based displacement measurement method that combines LiDAR and camera technologies. By applying coordinated transformations from image data to the world coordinate system, this method effectively calculates displacement using the obtained image and point cloud information, thereby compensating for measurement errors caused by out-of-plane motion. Hu et al. [95] introduced a method that combines MT InSAR and LiDAR data. They obtained key scatterers and parameters utilizing amplitude enhanced interferometry and correlated radar scatterers with point clouds through a 3D confidence ellipsoid. Experiments demonstrated a high degree of match between radar scatterers and laser points, highlighting significant classification improvements achieved through the integration of LiDAR data in radar scatterer classification.

In digital modeling and rendering of railroad bridges, in order to present railroad bridges with poles more accurately and realistically, Alexander [96] proposed a method to remove group self-noise from airborne LiDAR data by using a point-based PointPillars deep learning neural network to remove group self-noise from airborne LiDAR data, which was trained to detect and locate UAVs, successfully removing over 99% of the group self-noise, with no false positives when applied to a 7 million point LiDAR dataset. In addition, when applying BIM to an existing structure, it is an important task to construct a 3D model that accurately represents the as-built state and manually specify each structural component. Park [97] proposed a “Motion Semantic Architecture” method that utilizes the photo data from a UAV-mounted camera to construct a 3D model and automatically specifies the structural components, which was validated and tested in the case of a railroad bridge. In the validation test of a railroad bridge, the average accuracy, intersection ratio, and BF score reached 80.87%, 66.66%, and 56.33%, respectively, and successfully generated a 3D point cloud model reflecting the as-is states.

### 5.2. Tunnel Structure

In the field of railway tunnel engineering, the rapid development of urban rail transit networks—particularly the significant increase in the number of tunnels in China—has necessitated resolving a series of technical challenges related to tunnel detection, modeling, structural analysis, and safety. Many scholars have conducted extensive research in this field, yielding substantial results. Traditional methods for railway tunnel clearance inspection are characterized by low density and slow speeds. Zhou et al. [98] used a mobile laser scanning (MLS) system to capture 3D point clouds and proposed a new inspection method that encompasses the establishment of a dynamic coordinate system, extraction of railway lines, seamless connection of rail sections, and the implementation of a gap frame operation. The accuracy of this method can reach 0.03 m in tunnel testing applications, which enables effective clearance calculations. Yue et al. [99] addressed deficiencies in existing clearance convergence measurement methods, such as high manpower and equipment demands, the inability to measure the full section, low efficiency, and incomplete results, by proposing a tunnel section clearance convergence calculation method that is applicable to various parameters of shield and mined tunnels. Their method demonstrated a repeatability and absolute accuracy within ± 3 mm, thereby enhancing measurement efficiency by enabling full-section assessments. In addition, Lei et al. [100] introduced a novel smart obstacle avoidance system aimed at improving the anti-collision performance of train manipulators used for high-speed railway tunnel lining inspections. This system is capable of detecting obstacles as small as 0.5 cm in diameter, maintaining a mean control deviation between 1 and 2 cm, and allowing a 3 cm safety buffer. In contrast with conventional obstacle avoidance sensors, it delivers multi-dimensional information, which greatly facilitates the advancement of automated tunnel inspection management systems and railway tunnel digital twin lifecycle analysis technology.

**Figure 17:**
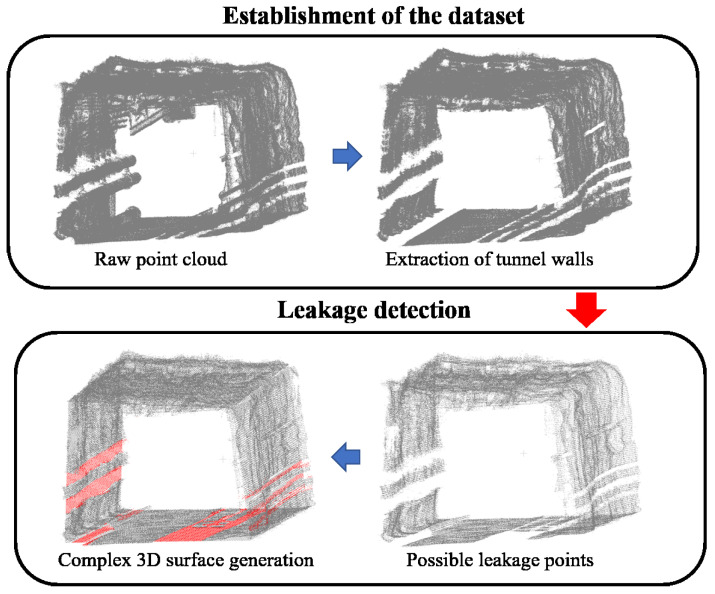
Leakage area extraction flowchart.

Although dynamic tunnel monitoring systems can collect cross-section data, the accuracy of odometer positioning alone may be insufficient, and the cross-section is typically limited to 3D displays within a local coordinate system. Du et al. [101] focused on improving relative positioning through MLS to improve the accuracy. They proposed a unified positioning method that links the cross-section to a broader system to meet both accuracy and 3D display requirements. Smith et al. [102] addressed the bending and deformation characteristics of masonry tunnel sections by proposing an automatic workflow capable of automatically identifying spalling depth from the 3D point cloud data obtained via laser radar. Hawley et al. [103] confirmed that the mobile LiDAR scanners can effectively detect leakage areas in underground tunnels, thereby offer an automatic workflow that extracts quantitative information about each leakage, as shown in Figure 17. Field tests demonstrated that this system can quickly capture, identify, extract, and record leakage areas based on the strength and spatial information derived from tunnel point cloud data, which significantly reduces measurement time and provides inspectors with a comprehensive 3D model of the tunnel accompanied by quantitative leakage information (location and area). Despite the improvements in deformation detection and defect identification of subway tunnels provided by mobile laser measurement systems, challenges such as high equipment costs and insufficient after-sales support persist. Li et al. [104] developed a mobile laser measurement system that integrates multiple modules such as track inspection vehicle and 3D laser scanner, achieving multi-sensor time synchronization. The reliability and stability of this system were validated through experiments conducted in the Xuzhou Metro. The operational safety of metro shield tunnels depends on timely deformation detection; however, numerous ancillary facilities and bolt holes can compromise detection accuracy. Shi et al. [17] introduced a cloth simulation filtering technique utilizing cylindrical projection of point clouds, which can effectively extract lining point clouds that satisfy the accuracy criteria for subway tunnel deformation monitoring.

**Figure 18:**
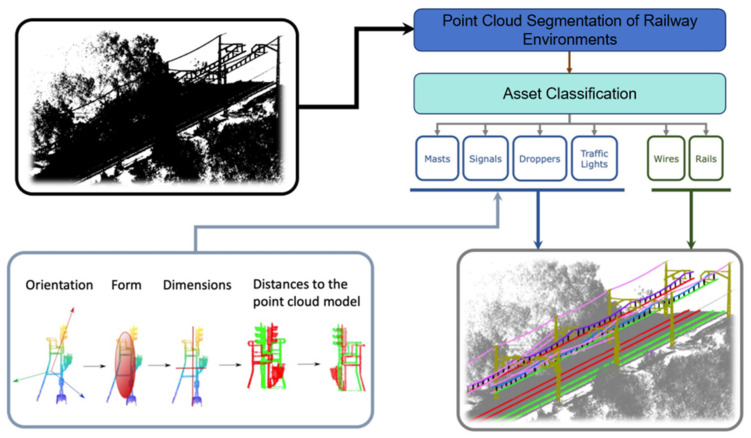
Semantic Segmentation Workflow for Complex Railroad Environments [108].

In point cloud processing, the lack of linear distribution and the distinct characteristics of railway tunnel structures presents challenges for 3D point cloud registration and positioning. Wang et al. [105] presented a global registration strategy founded on coordinates that demonstrates robustness and applicability in complex scenes, which facilitates the accurate positioning and registration of multi-station clouds in linear and curved railway tunnels. This method reduced the registration error by 65% compared to point-based registration techniques and achieved precision at the fine registration level. Li et al. [106] proposed a multi-layer classification method that utilizes a hierarchical clustering structure for processing underground tunnel point cloud data, which enables the gradual extraction of four types of ground objects, such as tracks and platforms. This method demonstrates high accuracy in both projection plane evaluation and point cloud classification, achieving an accuracy of 91.49% for the projection plane evaluation and 92.63% for the point cloud classification. To address the need for tunnel point cloud denoising without pretreatment, Bao et al. [107] developed a comprehensive treatment scheme that employs a Huber loss criterion to precisely estimate the geometric parameters of tunnel linings and perform point cloud noise reduction. They constructed a 3D coordinate system transformation method tailored to the characteristics of data distribution to address challenges associated with coordinate system transformations during point cloud noise reduction. Lamas et al. [108] proposed a heuristic-driven process for the semantic segmentation of intricate railway environment, as shown in Figure 18, which can classify the key components such as rails and masts. By leveraging existing point cloud analysis and partitioning methods combined with the shape and spatial context of each classification element, this workflow achieved notable success in classifying long and variable railway sections and facilitating auxiliary labeling of point cloud data for future applications based on the supervised learning model.

With the growing popularity of BIM, the demand for creating or updating existing tunnel original models has significantly increased. Farahani et al. [109] used 3D ground laser scanning instrument to scan tunnels in real environment for geometrical reconstruction. To address challenges posed by irregular rock surfaces and area loss from drilling and blasting, they constructed small-size point cloud model through additive manufacturing for laboratory testing and developed a laser scanning system to obtain the 3D tunnel model contours. Based on successful geometric document results, they proposed a real-scale 3D LSS model suitable for real-world applications. Cheng et al. [110] tackled the time-consuming and costly challenges of geometric modeling without design BIM by developing a general technology that employs ground laser scanning (TLS) data. By conducting point cloud classification and model parameter estimation, they created various classification algorithms based on geometric characteristics to identify different components, such as rails and cross-sections in monorail tunnels, thereby establishing a parameterized original BIM. Their findings demonstrated that rail, lining, and shelter models achieved millimeter-level accuracy, while pipeline and catenary equipment models achieved centimeter-level accuracy. Cheng et al. [111] developed a method that automatically segments instances of main, distributed, and curved rebars and reconstructs tunnel lining reinforcement from raw point clouds. This method includes five key components: reinforcement mesh remodeling, extraction optimization, semantic segmentation, instance marking, and modeling. The reconstructed reinforcement model achieved an accuracy level of approximately 2 mm.

Point cloud data have achieved remarkable advancements in the detection of railway bridges and tunnels, showcasing significant development potential. Currently, substantial progress has been made in structural deformation detection and monitoring of bridges and tunnels. By integrating real data, the fusion of models with actual measurements can achieve a transformative leap forward. In terms of tunnel detection, point cloud data enable accurate restoration of real scenes and the precise identification of issues such as cracks, water leakage, and lining detachment. In the future, bridge detection based on UAV images and point cloud data is expected to play a more prominent role in the visible detection of bridge diseases. Furthermore, integrating tunnel detection with DT technology enables real-time visualization of the disease progression, thereby facilitating timely interventions to prevent further deterioration and promoting the intelligent and efficient operation and maintenance of railway bridges and tunnels.

### 5.3. Disscussion and Key Technical Challenges in Railway Infrastructure

To provide clearer quantitative and comparative insights, Table 1 summarizes representative performance characteristics of point cloud-based methods across different railway infrastructure applications, based on commonly reported results in the literature.

Recent advances in railway infrastructure monitoring increasingly rely on the convergence of multiple disciplines. The integration of photogrammetry and LiDAR enables complementary data acquisition, while artificial intelligence techniques facilitate automated feature extraction and semantic understanding from large-scale point clouds. Moreover, the emergence of digital twin frameworks has further expanded the application scope of point cloud data, enabling continuous condition monitoring and system-level analysis. Such interdisciplinary approaches have significantly accelerated innovation in railway inspection, demonstrating the importance of collaborative research across sensing, data processing, and engineering application domains.

Despite the widespread application of point cloud technology in railway infrastructure monitoring, several fundamental challenges and bottlenecks limit its practical deployment and large-scale adoption. First, robustness of data acquisition remains a major challenge in complex railway environments characterized by varying lighting, occlusion, vibration, and dynamic operating conditions. These factors often result in incomplete or noisy point cloud data, particularly in scenarios such as tunnels, stations, and high-speed railways. Second, processing large-scale, high-density point cloud data incurs significant computational and storage burdens. While many existing methods demonstrate high accuracy, their efficiency and scalability are often insufficient to meet the demands of real-time or network-level railway monitoring. Third, generalization ability across different railway scenarios is generally limited. Methods developed for specific tasks or assets often experience performance degradation when applied to other infrastructure components, sensor configurations, or operating conditions. Finally, a gap remains between technological advancements and engineering decisions. Although point cloud-based methods can provide detailed geometric and semantic information, their direct integration with maintenance planning, asset management, and operational decision support systems remains largely unexplored.

It should be noted that progress in different railway application domains is highly interrelated. For instance, accurate track geometry extraction provides essential spatial references for foreign object detection, while high-resolution point cloud modeling forms the basis for predictive maintenance and digital twin development.

## 6. Conclusions and Prospect

Compared with traditional manual inspection or image-based methods, point cloud-based approaches generally report higher spatial resolution and improved detection reliability, albeit at the cost of increased computational complexity. This study summarizes the application of point cloud technology across the entire lifecycle of railway construction, operation, and maintenance. By enabling high-precision 3D reconstruction and intelligent analysis, point cloud technology is reshaping the paradigm for railway infrastructure inspection and maintenance. Current research has achieved sub-millimeter dynamic measurement of track geometry and, with advanced acquisition equipment, can detect subtle defects such as rail surface irregularities and bolt cracks. In the future, integrating point clouds with machine vision is expected to further enhance intelligent railway operation and maintenance. For catenary components, detection accuracy remains at the millimeter level due to train vibrations and acquisition distances; future work should target sub-millimeter or even micron-level wear detection and prediction. In railway environment monitoring and foreign object intrusion detection, progress has been made through geometric and parametric modeling of environmental elements and obstacles. In bridge and tunnel inspection, point clouds are used not only for structural deformation monitoring but also combined with UAV imagery for visual disease detection. The implementation of digital twin technology will further enable real-time tunnel condition monitoring, improving the effectiveness of structural health monitoring. Additionally, advances in deep learning and multimodal data fusion have enhanced target segmentation and feature extraction in complex environments, laying a technical foundation for intelligent railway maintenance. However, the current technology still faces challenges such as trade-offs between data acquisition efficiency and processing accuracy, conflicts between algorithm complexity and real-time requirements, and insufficient cross-platform data interoperability, which hinder its widespread engineering application. Recent advances in railway infrastructure monitoring increasingly rely on multidisciplinary integration. The convergence of photogrammetry and LiDAR enables complementary data acquisition, while artificial intelligence techniques facilitate automated feature extraction and semantic interpretation of large-scale point cloud data. Moreover, emerging digital twin frameworks further expand the application scope of point clouds by enabling continuous condition monitoring and system-level analysis.

From a system-level perspective, point cloud technologies are becoming a key enabler of railway digital transformation. By supporting high-fidelity infrastructure modeling and accurate condition assessment, point cloud-based approaches provide essential data foundations for automated inspection, intelligent asset management, and improved operational efficiency. As these technologies continue to mature, their integration into intelligent railway systems is expected to significantly enhance the safety, reliability, and sustainability of railway operations.

Future research needs to address three key technical challenges. Future efforts should focus on improving point cloud data quality, noise reduction, and efficient processing algorithms, particularly for operational and near-real-time inspection tasks. Research should increasingly emphasize multi-source data fusion, integrating point clouds with images, positioning data, and inertial measurements to enhance robustness and adaptability across diverse railway scenarios. In the long term, point cloud technologies are expected to serve as a foundational data layer for intelligent railway systems, supporting digital twins, predictive maintenance, and lifecycle-oriented asset management.

## Figures and Tables

**Table 1:** Representative quantitative and comparative characteristics of point cloud-based methods in railway infrastructure.

Application	Point Cloud Source	Typical Performance	Efficiency	Advantage over Traditional Methods
Track geometry extraction	Mobile LiDAR	mm–cm accuracy	Medium–High cost	Higher precision, full 3D geometry
Component detection (rails, sleepers et al.)	Mobile/TLS	>90% detection accuracy	Medium	Robust under occlusion
Tunnel & bridge modeling	Terrestrial LiDAR	cm/sub-cm resolution	High	High completeness
Surface defect detection	Dense LiDAR	+10–30% improvement	High	Fine surface details
Clearance & intrusion analysis	Mobile LiDAR	cm-level error	Medium	Reliable spatial measurement
Foreign object detection	Mobile LiDAR	High reliability (large objects)	Medium	Strong 3D discrimination
Condition monitoring	Long-term LiDAR	High repeatability	High	Enables degradation tracking
Predictive maintenance	Integrated point clouds	Improved planning efficiency	Medium–High	Data-driven decisions
Digital twin modeling	Multi-source point clouds	High geometric fidelity	High	System-level representation

## Data Availability

The author does not have permission to share the data.
